# Lectin-Directed Protein Aggregation Therapy toward Hyperfucosylated and Hypersialylated Metastatic Colorectal Cancers

**DOI:** 10.34133/bmr.0394

**Published:** 2026-07-17

**Authors:** Xiao Han, Yufei Li, Yiling Liu, Shenghai Shen, Yitong Shen, Li-Sheng Zhang, Kenward Vong

**Affiliations:** ^1^ Department of Chemistry, The Hong Kong University of Science and Technology, Hong Kong, China.; ^2^ Division of Life Science, The Hong Kong University of Science and Technology, Hong Kong, China.

## Abstract

Aberrant glycosylation is a well-known pathological alteration that accompanies tumor onset, progression, and eventual metastasis. For colorectal cancers, there is strong evidence to show that glycans containing α2,3-sialic acid, α2,6-sialic acid, and fucose are frequently up-regulated. In this work, we report the application of lectin-directed protein aggregation therapy (LPAT) to target hypersialylated and hyperfucosylated colorectal cancer cells. This system relies on the concept of cancer-activated lectin multivalency, where tumor-associated proteases can elicit the self-assembly of multivalent lectin complexes that can selectively impair the metastatic activities of cancer cells. After screening LPAT agents against a panel of 5 colorectal cancer cell lines, the most effective targeting was identified against the hyperfucosylated/matrix metalloproteinase-9-overexpressing HCT-116 cell line, which showed significant reductions in invasion and migration upon treatment. Experiments then showed that hyperfucosylation targeting could be used as a viable approach to prevent liver and kidney tumor development in a metastatic colorectal mouse model. Overall, this work highlights the viability of using LPAT to discriminate the aberrant glycosylation of highly metastatic colorectal cancer cells as a means to prevent their onset and progression.

## Introduction

As cancer is one of the leading causes of death worldwide, there has been considerable interest in developing targeting therapies that can selectively act to eradicate cancerous cells while sparing normal, healthy tissues [1]. This principle is the basis behind the engineering of monoclonal antibodies, which are therapeutics that act to target cancers through the recognition of specific cancer biomarkers [2]. Despite their success, antibody therapies have continually struggled with regard to targeting glycan-related cancer biomarkers.

Since the late 1960s, glycans present on the surface of cancer cells have been found to be markedly altered compared to those of normal cells. Deeper investigations revealed that tumor-associated glycans are characterized by changes in glycan composition, truncation, or modification of branching points [3]. These alterations were subsequently found to be linked to carcinogenesis, immune evasion, and even metastasis [4].

Two of the most well-known cancer-associated glycosylation changes found in cancers are hypersialylation and hyperfucosylation [5]. Sialic acid is a 9-carbon sugar mainly found as a terminal monosaccharide linked to other glycans through either α2,3- or α2,6-linkages. In cancers, hypersialylation has been linked to the up-regulation of sialyltransferases, which are enzymes that catalyze the transfer of sialic acid onto carbohydrate chains. For example, evidence has shown that ST3Gal variants (α2,3-sialyltransferase) are commonly up-regulated in hepatocellular carcinoma, ovarian cancer, and breast cancer. Likewise, ST6Gal variants (α2,6-sialyltransferase) have been found to be up-regulated in cancers found in the breasts, colon, and cervix.

Another glycan of interest is fucose, which is a 6-carbon hexose deoxy sugar found as 2 major types. Terminal fucosylation occurs when fucose is added to a terminal galactose by an α1,2-linkage or to a subterminal *N*-acetylglucosamine (GlcNAc) by an α1,3-linkage or an α1,4-linkage. Alternatively, core fucosylation occurs when fucose is added to the innermost GlcNAc of an *N*-glycan core by an α1,6-linkage. Numerous studies have directly linked hyperfucosylation with the up-regulation of fucosyltransferases (FUTs), which are responsible for catalyzing the addition of fucose onto carbohydrate chains. For example, high levels of fucosylation caused by up-regulated FUT1 and FUT2 has been detected in colon cancer [6,7], breast cancer [8], and lung adenocarcinoma [9].

Colorectal cancer (CRC), which develops from the colon or rectum, is one of the most common causes of cancer-related deaths worldwide [10]. The lethality of CRC is further highlighted by the fact that up to 50% of all patients will eventually develop metastatic tumors [11]. Given these facts, there is substantial interest in the development of targeted therapies against CRCs.

**Fig. 1.**
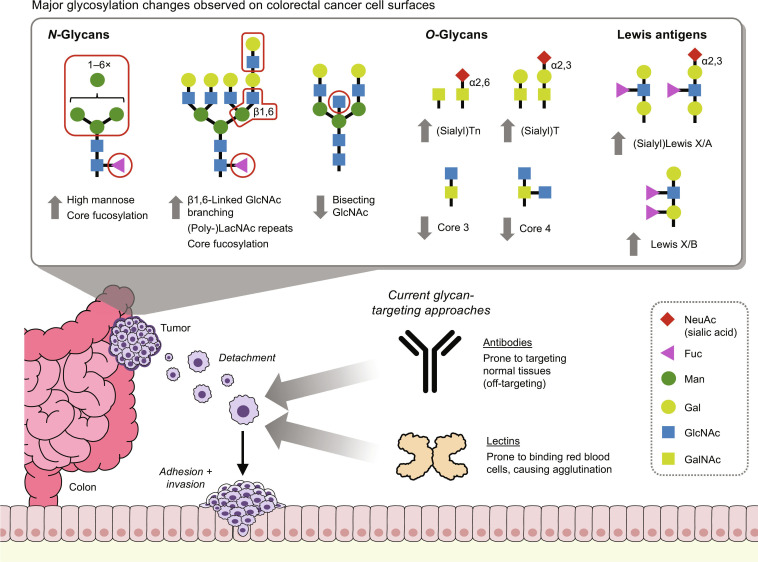
Aberrant glycosylation in colorectal cancer is a well-known phenomenon that results in changes that promote processes such as invasion, metastasis, and immune evasion. In general, studies have identified a general increase of glycan epitopes (i.e., *N*-glycans, *O*-glycans, and sialyl Lewis antigens) that increase overall levels of α2,3-sialic acid, α2,6-sialic acid, and fucose. With current technologies, researchers should be able to target cancer-associated glycans through approaches based on antibodies and lectins. It is reasoned that by targeting aberrantly glycosylated cancer cells, metastatic tumor spread can be prevented by inhibiting key cell adhesive processes. However, the usage of antibodies and lectins are also presented with their own unique disadvantages, such as undesired off-targeting and hemagglutination, respectively.

Without exception, CRCs are also prone to undergo aberrant glycosylation to facilitate tumor progression and survival. Outlined in Fig. 1 is a summary of the major glycosylation changes identified in CRCs. When looking at *N*-glycans, major changes include increased levels of high mannose [12], core fucosylation [13], β1,6-linked GlcNAc branching [14], and (poly-)LacNAc repeats [15]. A number of *O*-glycans have also been found to be greatly altered in CRCs. For example, elevated levels of (sialyl)T and (sialyl)Tn antigens found in malignant samples can be directly linked with the up-regulation of relevant sialyltransferases [16,17]. Likewise, sialyl Lewis X and sialyl Lewis A antigens are also shown to be elevated [18]. In the case of Lewis Y and Lewis B antigens, studies have tied their up-regulation to increased levels of FUTs [19]. Overall, major glycosylation changes in CRCs appear to generally increase glycans containing α2,3-sialic acid, α2,6-sialic acid, and/or fucose.

With the strong evidence for cancer-related hypersialylation and hyperfucosylation, there is an obvious inclination to develop targeted therapies that can utilize sialic acid and fucose as cancer biomarkers. While numerous antibody-based candidates have entered clinical trials throughout the past decades, they have seen very limited success. One reason for these failures is that all tumor-associated glycans are still principally composed of glycans of human origin. This exacerbates the issues of antibody off-targeting as normal cells can also produce sialic acid and fucose, albeit at lower levels. Another approach to glycan targeting has been the use of lectin-based therapies. Lectins are a large family of proteins capable of binding various glycan ligands in a specific and reversible nature. However, since lectins naturally required oligomerization to benefit from high-avidity binding (lectin multivalency), lectins are also known to suffer from the adverse side effects of hemagglutination. Due to this danger, no lectin-based targeted therapeutics have ever progressed past clinical trials.

**Fig. 2.**
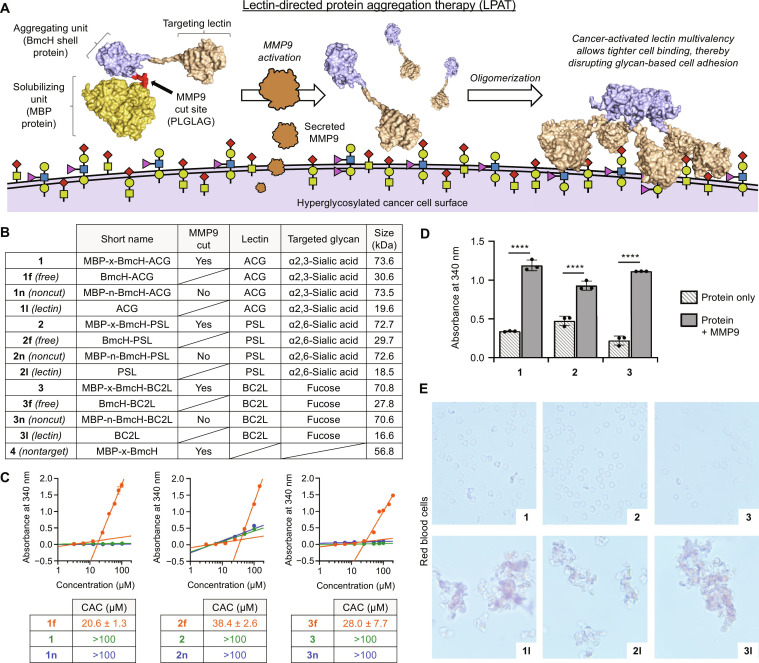
(A) The design of lectin-directed protein aggregation therapy (LPAT) uses cancer-specific proteases and controllable lectin multivalency to allow for selective binding to the hypersialylated and hyperfucosylated surfaces of colorectal cancer cells. After removal of the solubilizing unit by matrix metalloproteinase-9 (MMP9), activated LPAT monomers can oligomerize to create tight-binding protein complexes for their targeted glycans. (B) Table of the LPAT agents prepared for this study. LPAT agents 1 to 3 were designed specifically to possess varying sialic acid- and fucose-binding lectins. The following LPAT analogs were designed as controls; 1f to 3f lacked a solubilizing unit to prove oligomerization in vitro, 1n to 3n had a random cut site to prove MMP9 activation, and 4 lacked any targeting lectin moiety to prove biological effects were caused by glycan binding. (C) To probe oligomerization potential, turbidity plots were acquired for LPAT agents 1 to 3, solubilizing-unit-lacking 1f to 3f, and uncleavable 1n to 3n. The presented critical aggregation concentrations (CACs) were then determined based on these plots. (D) To confirm MMP9 responsiveness that can lead to self-assembly/protein aggregation, LPAT agents 1 to 3 (100 μM) were incubated with or without MMP9 (0.1 μg/ml) for 24 h. Turbidity (at 340 nm) was then quantified, which can correlate with protein aggregation. (E) When LPAT agents 1 to 3 were incubated with a 1% red blood cell (RBC) solution for 1 h at room temperature, microscope images (at 40× magnification) showed that they lacked any hemagglutination activity. Under similar conditions, lectin-only 1l to 3l were clearly shown to induce blood cell agglutination. Statistical analysis was performed using an unpaired *t* test. All numerical data are presented as mean ± standard error of the mean (SEM) of 3 replicates. *P < 0.03; **P < 0.002; ***P < 0.0002; ****P < 0.0001; ns, not significant.

Despite the current limitations in glycan-targeting technologies, enthusiasm for creating alternative glycan-targeting mechanisms has not dampened [20–24]. In this regard, our group has recently developed the concept of lectin-directed protein aggregation therapy (LPAT) [25]. These therapeutic agents were created with 4 main components: a glycan-targeting lectin, an aggregating protein unit, a linker region with a protease responsive cut site, and a solubilizing protein unit (Fig. 2A). This system exploits principles of lectin multivalency and cancer-specific proteases to ensure that LPAT agents accumulate mainly onto surfaces of cancer cells, rather than normal cells. It does this through a dual-targeting approach where overexpressed matrix metalloproteinases (MMPs) on cancer cells are first needed to cleave off the solubilizing unit. This action frees the aggregating protein unit to regain its self-assembling properties. Due to hyperglycosylation, a higher localized concentration of LPAT agents then form on cancer cell surfaces, thereby promoting oligomerization that increases binding (via lectin multivalency), leading to impaired cell adhesion, invasion, and migration. This strategy was previously shown to be successful in impairing the metastatic properties of hypersialylated breast cancer cells [25].

In this study, the aim was to design and develop LPAT agents that can target and impair the metastatic properties of CRC, which is known to have up-regulated levels of α2,3-sialic acid, α2,6-sialic acid, and fucose. To do this, various LPAT agents with different lectin-targeting moieties were created and then screened against a panel of CRC cells.

## Materials and Methods

### Recombinant protein expression and purification

Plasmids containing the genes of the LPAT agents used in this study were created by inserting relevant DNA fragments (obtained via synthesis) into pET-21a(+) vectors through a combination of NdeI, BamHI, HindIII, and XhoI cut sites. To enable the bacterial expression of these plasmids, transformations were performed using commercially available One Shot BL21(DE3) Chemically Competent *Escherichia coli* (Thermo Fisher). Transformed bacteria were generally grown in Luria–Bertani media supplemented with ampicillin (50 μg/ml). To induce protein expression, overnight bacterial cultures were used to inoculate larger Luria–Bertani cultures (500 ml), which were then placed in shaking incubators at 37 °C. At an optical density reading of 0.6, isopropyl β-d-1-thiogalactopyranoside was added to a final concentration of 0.5 mM and the cultures were then grown for 4 h at 28 °C. To carry out the extraction of proteins, the collected bacterial pellets were resuspended in lysis buffer (20 mM tris, 300 mM NaCl, and 1 mM phenylmethanesulfonyl fluoride, pH 7.4) supplemented with protease inhibitors. The cell suspension was then sonicated (5 s on/10 s off for 15 min) and centrifuged (12,000 rpm at 4 °C for 20 min) to obtain the supernatant. Protein purification was carried out by loading the supernatant onto a HisPur Ni-NTA cartridge (Thermo Fisher) connected to an ÄKTA start fast protein liquid chromatography system (Cytiva). To elute the desired LPAT protein, an imidazole gradient (0 to 300 mM) was slowly applied to the column. After confirmation of the eluted protein fractions by sodium dodecyl sulfate–polyacrylamide gel electrophoresis, desired LPAT proteins were collected and combined. Using 30-kDa molecular weight cut-off protein concentrators, the volume of the protein solutions was reduced and buffer exchange was performed.

### Hemagglutination assay

The assay was carried out as previously described [25]. Whole blood was collected from a healthy male volunteer and immediately mixed with 3.8% sodium citrate solution in a 1:1 ratio. The mixture was then centrifuged at 2,000 g for 4 min at 4 °C. The supernatant was carefully discarded, followed by washing the cell pellet with 3 washes with 100 μl of saline. The pellet of red blood cells was then diluted to 2% (v/v) in saline and tested immediately. To perform the agglutination assay, 50 μl of LPAT **1** to **3** or **1l** to **3l** stock solutions (20 μM) was incubated with 50 μl of the 2% red blood cell solution. Following incubation for 1 h at room temperature, samples were agitated and 10 μl was pipetted onto a chamber slide. Using a trinocular microscope equipped with a 10-megapixel camera (AmScope), images of the red blood cells were obtained at 10 × and 40 × magnifications.

### Cell culture conditions

In this study, the human colon cancer cell lines DLD-1, HT-29, SW620, HCT-116, and LoVo were obtained from the Japanese Collection of Research Bioresources cell bank (Japan). The normal colon cell line NCM-460 was acquired from iCell Bioscience (China). HCT-116 and HT-29 were maintained in McCoy’s 5A media (Gibco) with 10% fetal bovine serum (FBS) (ExCell Bio); SW620, DLD-1, and NCM-460 were maintained in RPMI 1640 media with 10% FBS, while LoVo was maintained in Ham’s F12K media with 20% FBS. All media were additionally supplemented with 100 μg/ml of penicillin–streptomycin (Gibco). Cells were grown in an incubator set at a temperature of 37 °C with an atmosphere of 5% CO_2_.

### Measuring cell surface glycosylation (via metabolic labeling)

The total sialic acid and fucose content on cell surfaces was measured using metabolic labeling. To start the assay, 5 × 10^5^ of relevant cells were seeded onto 6-well plates with at least 2 ml of appropriate growth media. These plates were then incubated overnight at 37 °C. For sialic acid measurements, cells were given fresh media either with or without 40 μM tetraacetylated *N*-azidoacetylmannosamine (Ac_4_ManNAz; Lumiprobe). After an incubation period of 48 h, the cell medium was replaced with 2 ml of 10 μM dibenzocyclooctyne (DBCO)–fluorescein (Lumiprobe) in phosphate-buffered saline (PBS) with 5% FBS. For fucose measurements, cells were given fresh media with or without 400 μM peracetylated 6-azidofucose (Ac_4_6AzFuc; Apollo Scientific). Following an incubation period of 48 h, the cell medium was replaced with 2 ml of 20 μM DBCO–fluorescein (Lumiprobe) in PBS with 5% FBS. With this DBCO–fluorescein containing medium, cells were incubated for an additional 1 h at 37 °C. To prepare for the next step, adherent cells were washed 4 times with PBS buffer. Cells were then harvested by trypsinization and then collected by centrifugation. After preparing a ~1 × 10^6^ cell suspension in 1 ml of fluorescence-activated cell sorting (FACS) buffer (2% FBS in PBS), FACS was performed using FACSAria III Cell Sorter (BD). Gating was set to 10,000 events using a fluorescein isothiocyanate (FITC) channel (250 V for sialic acid and 350 V for fucose). Other instrument settings include setting the filter at 2.0, the nozzle at 85 μm, and the flow rate at 1.0 ml/min. Histograms and their mean fluorescence intensity (MFI) were generated by the FCS Express 7.22 software.

### Measuring cell surface glycosylation (via lectin binding)

Experiments were modified from literature protocols [26,27]. The differential expression of α2,3- and α2,6-linked sialic acid was measured in binding studies utilizing *Maackia amurensis* lectin I (MAL I) and *Sambucus nigra* lectin (SNA), respectively. A freshly trypsinized suspension of 1 × 10^6^ cells was placed in a 15-ml conical tube. The cells were then washed twice: centrifugation (210 rcf for 3 min), removal of supernatant, and resuspension of the pellet in PBS buffer. For the first step, cells were incubated with a 100-μl solution of either 10 μg/ml SNA–biotin (Vector Labs) in PBS, 10 μg/ml MAL I–biotin (Vector Labs) in PBS, or PBS only as a control. Cells were gently shaken for 30 min at 4 °C and then washed twice in a manner similar to the one previously described. For the second step, cells were incubated with a 100-μl solution of 0.3 μg/ml streptavidin–FITC (eBioscience) in PBS. Cells were gently shaken for 20 min at 4 °C in the absence of light. Finally, cells were washed 3 times similar to the manner previously described. Following centrifugation, a ~1 × 10^6^ of cell suspension in 1 ml of FACS buffer (2% FBS in PBS) was prepared. FACS was carried out using FACSAria III Cell Sorter (BD). Gating was set to 10,000 events using an FITC channel (350 V). Other instrument settings include setting the filter at 2.0, the nozzle at 85 μm, and the flow rate at 1.0 ml/min. Histograms and their MFI were generated by the FCS Express 7.22 software.

### Measuring MMP9 levels via ELISA

MMP9 secreted among the various cancer cell lines in this study were quantified using a human MMP9 enzyme-linked immunosorbent assay (ELISA) kit (Excell Bio). To perform this assay, 2 × 10^6^ cells of each cell line were first seeded and grown overnight at 37 °C on 10-cm culture plates. Afterward, the medium was replaced with 10 ml of serum-free growth media. After an incubation period of 2 d at 37 °C, the growth medium from each plate was collected and concentrated to a volume of 330 μl. To perform ELISA, 100 μl of the concentrated growth medium was placed into a well of the supplied 96-well microplate precoated with the capture antibody. After a period of 90 min, incubations with the antibody–biotin and streptavidin–horseradish peroxidase conjugates were carried out. Finally, tetramethylbenzidine was added to each well in order for the analyte levels to be measured via absorbance at 450 nm using VANTAstar Microplate Reader (BMG). Final analyte concentration levels were determined through extrapolation from a standard curve constructed using known levels of MMP9.

### Cytotoxicity determination

To determine cell viability, a commercial MTS assay kit (Abcam) was employed. For the short-term assay, cancer cells were first seeded onto 96-well plates at varying densities (1 × 10^4^ cells/well for HT-29 or 2 × 10^4^ cells/well for HCT-116, LoVo, SW620, and DLD-1). Following overnight incubation at 37 °C, wells were refreshed with 80 μl of media and 20 μl of LPAT agents **1** to **3** at different concentrations. After a 1-d incubation period, wells were then refreshed with 80 μl of media and 20 μl of the MTS reagent. After a 2-h incubation at 37 °C, cell viability could be measured by absorbance at 490 nm using VANTAstar Microplate Reader (BMG). For the long-term assay, cancer cells were first seeded onto 7 separate 96-well plates at a density of 1 × 10^3^ cells/well. Following overnight incubation at 37 °C, wells were refreshed with 80 μl of media and 20 μl of LPAT agents **1** to **3** at a final concentration of 10 μM. From days 3 to 9, wells from one chosen plate were all refreshed with 80 μl of media and 20 μl of MTS reagent. After a 2-h incubation at 37 °C, cell viability could be measured by absorbance at 490 nm using VANTAstar Microplate Reader (BMG).

### Cell adhesion assay using FBS-coated plates

This assay works by testing the capacity of cells to bind to the extracellular matrix proteins found in FBS. To start, 96-well assay plates were precoated overnight by incubation with relevant growth media supplemented with 10% FBS. In a separate 6-well plate, cells were seeded at a density of 3 × 10^5^ cells per well. Following overnight incubation at 37 °C, wells were refreshed with 400 μl of media and 100 μl of LPAT agents **1** to **3** or relevant controls (10 μM). After an incubation period of 24 h, cells were harvested and resuspended in serum-free media at specific concentrations (5 × 10^5^ cells/ml for HCT-116 and NCM-460 and 8 × 10^5^ cells/ml for SW620, DLD-1, LoVo and HT-29). To carry out the adhesion assay, 100 μl of each cell suspension was first placed in the individual FBS-coated wells. After incubating at 37 °C for different time points (2 h for HCT-116 and NCM-460; 3 h for SW620, DLD-1, and HT-29, and 4 h for LoVo), the medium was then carefully removed and any adherent cells were washed (4×) using PBS buffer. The presence of adherent cells was then quantified using a commercial MTS assay kit (Abcam).

### Cell adhesion assay using selectin-coated plates

This assay works by testing the capacity of cells to bind to relevant selectin proteins, which are important for the metastatic process. To start, Nunc MaxiSorp 96-well microtiter plates were precoated overnight at 4 °C by incubation with 100 μl of either a 3 μg/ml of human P- or E-selectin (Sino Biological) solution in assay buffer (20 mM Hepes, 150 mM NaCl, and 10 mM CaCl_2_, pH 7.4). The plates were washed with assay buffer (3×) and then blocked with a 3% w/v solution of bovine serum albumin in assay buffer for 3 h at 4 °C. To finish the plate preparations, wells were washed with assay buffer (3×) before usage. In a separate 6-well plate, cells were seeded at a density of 3 × 10^5^ cells per well. Following overnight incubation at 37 °C, wells were refreshed with 400 μl of media and 100 μl of LPAT agents **1** to **3** or relevant controls (10 μM). After an incubation period of 24 h, cells were harvested and resuspended in serum-free media to a concentration of 5 × 10^5^ cells/ml. To carry out the adhesion assay, 100 μl of cell suspension was first placed in the individual selectin-coated wells. After incubating at 37 °C for 2 h, the medium was then carefully removed and any adherent cells were washed (4×) using PBS buffer. The presence of adherent cells was then quantified using a commercial MTS assay kit (Abcam).

### Cell migration (wound healing) assay

This assay works by testing the migratory capacity of cells. To start, 24-well plates were seeded with relevant cells and the cells were grown to about 90% confluency. A gap was made to the monolayer of cells by scratching with a sterile micropipette tip. The cellular debris produced by this process was washed with PBS. Wells were then refreshed with 80 μl of media with 2% FBS and 20 μl of LPAT agents **1** to **3** or relevant controls (10 μM). Plates were then placed back into an incubator at 37 °C. The migratory activity of cells was observed using a Cell Discoverer 7 microscope (Zeiss) at a magnification of 5×. Photographs were taken at different time intervals (0, 24, 36, and 48 h) after LPAT treatment. Determination of the wound closure percentages was carried out using the ImageJ software.

### Cell invasion assay

This assay works by testing the invasive capacity of cells. To carry out this assay, Millicell Cell Culture Inserts (Merck Millipore) with an 8.0-μm pore size were acquired. These inserts were initially coated with Matrigel (1 mg/ml) and dried at 37 °C for 2 h. In a 6-well plate, cells were seeded at a density of 3 × 10^5^ cells per well. Following overnight incubation at 37 °C, wells were refreshed with 80 μl of media and 20 μl of LPAT agents **1** to **3** or relevant controls (10 μM). After an incubation period of 24 h at 37 °C, cells were harvested and resuspended in serum-free media. To carry out the invasion assay, approximately 1 × 10^5^ cells were pipetted into the hanging insert. Conversely, the lower chamber was filled with relevant growth media supplemented with 10% FBS. After incubating at 37 °C for 48 h, cells on the upper side of the insert membrane were considered noninvasive and removed using a cotton swab. Cells on the lower side of the insert membrane were considered invasive and were fixed/stained with 2% crystal violet in ethanol. The invasive activity of cells was observed using a trinocular microscope equipped with a 10-megapixel camera (AmScope) at a magnification of 10×. Determination of the number of invasive cells was carried out using the ImageJ software.

### Animal study conditions

To conduct animal studies according to the Animal Ethics Committee at the Hong Kong University of Science and Technology (HKUST), protocol AEP-2023-0065 was created, which operates in accordance with both institutional and Hong Kong guidelines. The parental lineage of the mice used in this study are from NOD scid gamma (NSG) mice (The Jackson Laboratory). Mouse husbandry followed strict pathogen-free procedures in the Laboratory Animal Facility (Clear Water Bay) facilities at HKUST. During experimentation, isoflurane inhalation was used as the method of anesthesia. Following experiment completion, euthanasia was performed according to carbon dioxide (CO_2_) inhalation protocols.

### Measuring anti-metastatic activity in mice (via an experimental metastasis model)

To carry out these experiments, female NSG mice (4 to 5 weeks of age) were used. To start, a solution of 1 × 10^5^ HCT-116 cells in 50 μl of sterile Hank’s balanced salt solution buffer was injected into each mouse tail vein. Mice were then separated into 2 groups randomly: a control group and a treatment group. For the control group, mice received 50 μl of a saline solution via tail vein injection. For the treatment group, mice were given 50 μl of LPAT **3** in saline (at a dosage of 12 mg/kg) via tail vein injection. After a period of roughly 8 weeks, the experiment was stopped by euthanization of the mice. Animal carcasses were then autopsied for tumor burden specifically on liver and kidney tissues. These organs were initially extracted and photographed, followed by the excision of their individual tumors. Tumors were roughly measured by a caliper before grouping and counting. All tumors were weighed and then frozen for hematoxylin and eosin (H&E) staining.

### H&E staining protocol

Fresh liver and kidney tissues were fixed in 4% formaldehyde for 24 h at 4 °C. Tissues were then thoroughly rinsed in PBS buffer, followed by gradient dehydration with 15% and 30% sucrose solutions. The tissues were then embedded in an optimal cutting temperature compound and rapidly frozen. Sections were sliced at a thickness of 5 μm using an NX70 cryostat (Thermo) and then mounted onto glass slides. For histological analysis, tissue sections were stained with an H&E kit (Solarbio). Sections were stained with hematoxylin for 3 min, rinsed in water, and blued in 0.1% ammonium hydroxide for 5 s. Counterstaining was performed with eosin Y for 15 s. Sections were finally dehydrated through a graded ethanol series (70%, 95%, and 100%), cleared in xylene, and then coated with neutral balsam.

### Gene expression level analyses

Comparative expression profiling of genes related to fucosylation was carried out between paired clinical isolates. To do this, RNA sequencing datasets were sourced from several studies [28–30], which were retrieved from their Gene Expression Omnibus repositories (GSE50760, GSE245351, and GSE132226). In total, data from 29 patients were deemed suitable for analysis due to the existence of paired data between their primary colorectal tumors and their metastatic tumors (liver or brain).

## Results and Discussion

To begin this study, LPAT agents **1** to **3** were designed as described in Fig. 2B. As previously optimized [25], the best solubilizing unit to employ is the bulky maltose-binding protein, which can be removed with an MMP9-specific cleavage sequence (PLGLAG). In addition, an aggregating unit based off the bacterial microcompartment protein BmcH was found to be the most responsive. For the lectin-based targeting units, 3 different proteins were explored. LPAT agent **1** uses the ACG lectin, which is derived from *Agrocybe cylindracea* and primarily recognizes α2,3-linked sialic acids [31]. LPAT agent **2** uses the PSL lectin, which is derived from *Polyporus squamosus* and chiefly recognizes α2,6-linked sialic acids [32]. Finally, LPAT agent **3** uses a lectin known as rBC2LCN, which is the N-terminal domain of the BC2L-C lectin from *Burkholderia cenocepacia* [33]. Studies have shown that rBC2LCN has a preference for α1,2-linked terminal fucose motifs. To act as mechanistic controls, a number of LPAT analogs were also included in this study. Proteins **1f** to **3f** were designed to lack the solubilizing unit so that the BmcH oligomerization potential could be evaluated in vitro. Proteins **1n** to **3n** were instead created with a random sequence (GFVGGS) instead of the MMP9 cut site. The intent of **1n** to **3n** was to serve as a negative control during functional tests to prove the importance of MMP9 activation. Finally, by testing the lectin-deficient LPAT **4**, observed biological effects can be confirmed to be caused directly by glycan binding.

Since LPAT agents **1** and **2** have been previously described [25], their aggregation propensities following MMP9 exposure were already well characterized. To ensure that LPAT agent **3** operates in a similar manner, critical aggregation concentration studies were carried out. Since **3f** is designed without a solubilizing unit, it has the ability to freely self-assemble in solution. Thus, the critical aggregation concentration of **3f** was determined to be roughly 28 μM, which is similar to the values previously obtained for **1f** and **2f** (Fig. 2C). However, whenever LPAT agents possessed a solubilizing unit, such as LPAT **1** to **3** and the uncleavable **1n** to **3n** controls, these proteins remained soluble even at concentrations up to 100 μM.

To next confirm MMP9 responsiveness, LPAT agents **1** to **3** were all incubated with activated MMP9 over 24 h and then assessed for aggregation (Fig. 2D). From these data, BmcH-based LPAT agents **1** to **3** were all shown to produce significant gains in turbidity, thereby proving that MMP9 triggered self-assembly and aggregation.

One important test in this study is whether the LPAT agents possess any adverse hemagglutination activity. To investigate this, a 1% red blood cell suspension was incubated with LPAT agents **1** to **3** for 1 h. Through observation (via a microscope), it was clear to see that red blood cells incubated with all LPAT agents remained separated and healthy (Fig. 2E). This is in contrast to when lectins **1l** to **3l** were tested under similar conditions, which clearly showed the formation of blood cell agglutinates.

**Fig. 3.**
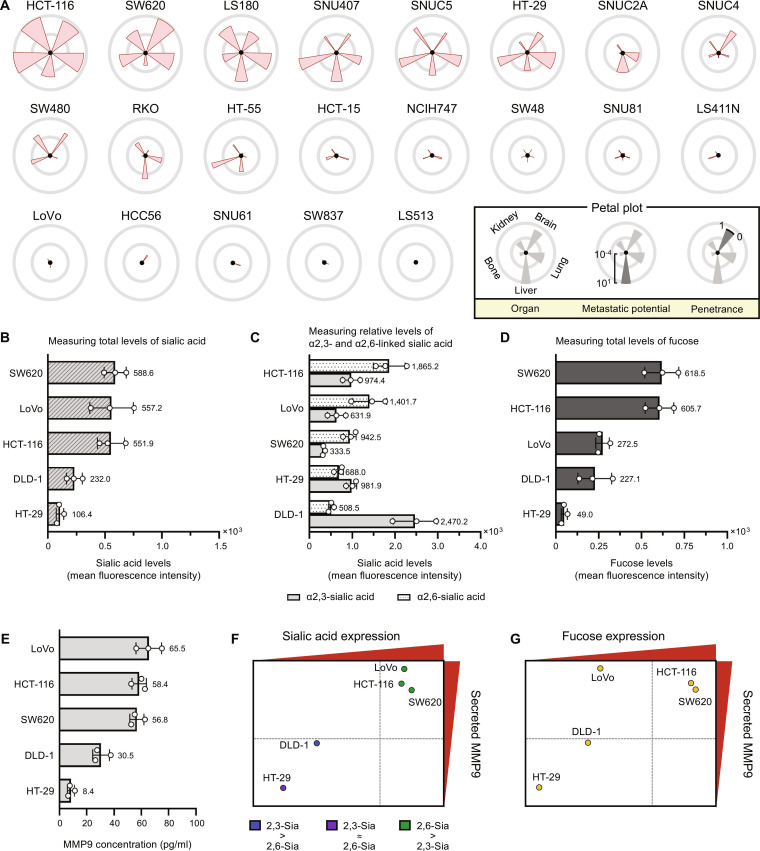
(A) Petal plots were constructed to examine the metastatic patterns of 21 colorectal cancer cell lines. Petal length represents metastatic potential, while petal width represents penetrance. For this study, profiling of the relative glycosylation and matrix metalloproteinase-9 (MMP9) expression levels in colorectal cancer cells was performed. (B) The total sialic acid content of cells was measured by metabolic labeling of cells incorporated with azide-containing sialic acid (via Ac_4_ManNAz), followed by incubation with dibenzocyclooctyne (DBCO)–fluorescein. (C) Lectin-based labeling studies were carried out to determine the relative expression levels between α2,3- and α2,6-linked sialic acids. Cells were incubated with either MAL I–biotin (α2,3-targeting) or SNA–biotin (α2,6-targeting), followed by incubation with streptavidin–fluorescein isothiocyanate (FITC). (D) The total fucose content of cells was measured by metabolic labeling of cells incorporated with azide-containing fucose (via Ac_4_6AzFuc), followed by incubation with DBCO–fluorescein. (E) The secreted levels of MMP9 for each cell line were determined via an enzyme-linked immunosorbent assay (ELISA) on the concentrated culture media following a 2-d growth period. (F and G) Summary charts were constructed to compare the relative balance between sialic acid or fucose expression and secreted MMP9 activities among varying colorectal cancer cell lines. All numerical data are presented as the mean ± standard error of the mean (SEM) of 3 replicates.

Moving on in this study, a crucial consideration is the choice of CRC cell lines to be tested. While there are likely many factors that contribute to the metastatic potential of tumor cells, an important question of LPAT is whether targeting hyperglycosylation/MMP9 overexpression can impair the metastatic process. To help identify the most metastatic CRC cell lines for testing, analysis was done on data obtained from a study that previously built a comprehensive metastasis map (MetMap) [34]. By using an in vivo barcoding strategy, this study tested the metastatic potential of numerous human cancer cell lines in mouse xenografts at scale. When specifically looking at CRCs (Fig. 3A), analysis showed that the HCT-116 and SW620 cell lines possessed the highest metastatic potential out of 27 tested cell lines. It should be noted that while the DLD-1 cell line is not included in this dataset, it is known in the literature that the metastatic potential of DLD-1 is poor enough to require external stimulus for testing [35]. Considering this analysis and available resources, this study chose to push forward with LPAT testing against the SW620, HCT-116, LoVo, DLD-1, and HT-29 CRC cell lines. However, before functional testing could begin, experiments were performed to characterize the glycan and MMP9 expression profiles of the cancer cell panel.

Beginning with sialic acid, total levels were quantified and compared through metabolic labeling (Fig. 3B). This can be done by cellular incorporation of Ac_4_ManNAz so that surface sialoglycoproteins are decorated with azides. Following DBCO–fluorescein labeling, cells are then analyzed by FACS to determine the MFI per cell. From these data, it can be observed that HCT-116, LoVo, and SW620 all express similar levels of sialic acid, which are generally higher than those of the HT-29 and DLD-1 cell lines. Although there is no literature precedence in comparing these cell lines through metabolic labeling, these data do corroborate with studies that have individually shown high sialylation levels in HCT-116 and LoVo [36,37].

Given the approach of using 2 different LPAT agents with differential sialic acid targeting (ACG for α2,3-sialic acid and PSL for α2,6-sialic acid), it is also necessary to investigate the ratio of α2,3- and α2,6-linked sialic acid for each cell line (Fig. 3C). To do this, biotinylated MAL I (α2,3-targeting) and SNA (α2,6-targeting) lectins were incubated with each cell line, followed by their detection via streptavidin–FITC addition. From these data, HCT-116, LoVo, and SW620 appeared to express higher levels of α2,6-linked sialic acid (1.9- to 2.8-fold), while DLD-1 alternatively displayed a higher level of α2,3-linked sialic acid (4.8-fold). Interestingly, HT-29 produced equal levels of α2,3- and α2,6-linked sialic acid (within error). This will likely make HT-29 difficult to discriminate between the 2 types of sialic acid-targeting lectins used in this study.

In the final glycan profiling experiment, metabolic labeling was performed to determine total fucose levels among the CRC cells (Fig. 3D). Through the cellular incorporation of Ac_4_6AzFuc, fucose-linked surface proteins were decorated with azides, thereby allowing for detection via click chemistry. From these data, the group of HCT-116 and SW620 showed the highest levels of fucose, followed by the group of LoVo and DLD-1 with moderate expression and then HT-29 with the lowest levels of fucose production. All acquired flow cytometry data related to glycan profiling are shown in Fig. S1.

Another cell characteristic crucial to the targeting functionality of LPAT is the overexpression and secretion of MMP9. Thus, an ELISA was next carried out on the concentrated culture media collected from each cell line following 2 d of growth (Fig. 3E). From these comparative data, LoVo, HCT-116, and SW620 all displayed the highest levels of detected MMP9 (56 to 65 pg/ml) compared to the other tested cell lines. This trend corroborates well with other literature sources, as these exact cell lines have been previously identified with high levels of MMP9 messenger RNA and protein amounts [38].

Overall, the collected data give an in-depth understanding of the glycan and MMP9 expression profiles for the CRCs chosen for this study. As a visual aide, summary charts highlighting the balance between glycosylation and MMP9 activities are shown in Fig. 3F and G. In general, it appears that HCT-116, SW620, and LoVo consistently display the most sialylation (or fucosylation) along with exhibiting the most MMP9 secretion. This is in contrast to HT-29 and DLD-1, which unfortunately do not fit the glycosylation and MMP9 profiles deemed necessary to be effectively targeted by LPAT.

One observed trend of note from the glycan and MMP9 expression profile data is the differential expression of α2,3- and α2,6-linked sialic acids among the cell lines. When looking at the cell lines with the highest sialic acid expression levels (LoVo, HCT-116, and SW620), these typically produce significantly more α2,6-linked sialic acid. However, with the lower-sialic acid-expressing cell lines (DLD-1 and HT-29), a greater preference is instead found toward α2,3-linked sialic acid expression. With the additional disparity in MMP9 expression between these 2 groups, this may appear to suggest that CRC cell lines with higher invasive properties end up producing higher levels of glycoproteins decorated with α2,6-linked sialic acids.

As one final note related to cancer cell line profiling, it is also important to be aware of the origins of the cell lines under study. According to the Dukes staging system for CRC, cell lines that fall under Dukes type C classification are characterized only by invasion into nearby lymph nodes (DLD-1, HT-29, LoVo, and SW620), but Dukes type D classification is characterized by cancers exhibiting widespread metastasis (HCT-116).

As previously described [25], LPAT was first shown to effectively impair the metastatic properties of hypersialylated breast cancer cells. Therefore, the focus of this study next turned to testing the biological activity of LPAT agents **1** to **3** against the CRC cells under study. First, short-term viability assays were run using **1** to **3** to confirm their noncytotoxic nature at concentrations up to 10 μM (Fig. S2). These data were further supplemented with long-term tests, where incubations over a 7-d period did not result in significant decreases in cell viability compared to that of controls (Fig. S3). Overall, these tests confirm that any biological activity from subsequent assays is likely to be caused by the cell surface binding of the MMP9-activated LPAT protein complexes, rather than an unknown pathway caused by a cytotoxic mechanism.

**Fig. 4.**
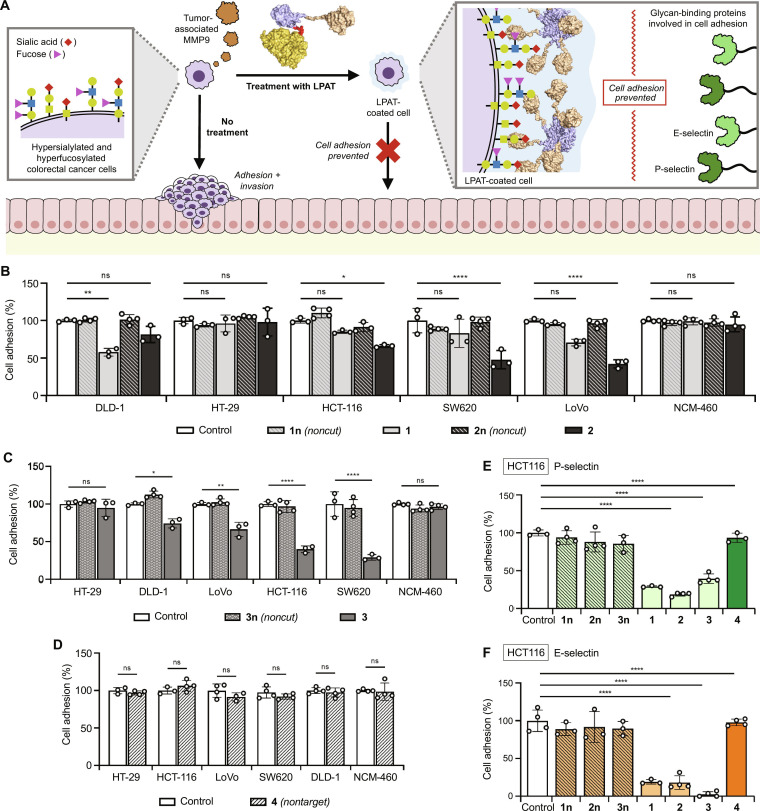
Antiadhesive properties of lectin-directed protein aggregation therapy (LPAT) agents 1 to 3. (A) It is hypothesized that once LPAT agents bind strongly to cell surfaces, the adhesive properties of colorectal cancer cells will be suppressed, thereby limiting their interactions with glycan-binding proteins (i.e., P- and E-selectin) to reduce their metastatic potential. Adhesion assays were performed using fetal bovine serum (FBS)-coated plates to test the inhibitory effects of (B) sialic acid-targeting LPAT agents 1 and 2 (10 μM), (C) fucose-targeting LPAT agent 3 (10 μM), and (D) nontargeting LPAT control 4 (10 μM) against varying colorectal cancer cell lines (DLD-1, HT-29, HCT-116, SW620, and LoVo) and one normal colon cell line (NCM-460). To investigate the effects to selectin-based binding, adhesion assays were done using HCT-116 cells exposed to LPAT agents 1 to 3 and their related controls (10 μM). The experiments were conducted using either (E) P-selectin-coated plates or (F) E-selectin-coated plates. All numerical data are presented as a mean ± standard error of the mean (SEM) of 3 replicates. Statistical analysis was performed using a one-way analysis of variance (ANOVA) with Tukey’s multiple comparisons test. *P < 0.03; **P < 0.002; ***P < 0.0002; ****P < 0.0001; ns, not significant.

In the concept of LPAT as an anti-metastatic agent, cell binding of the protein complexes is meant to occupy crucial glycan-binding sites that are necessary for selectin-based adhesion. Selectins are a family of transmembrane glycan-binding proteins that are primarily involved in cell adhesion. Two well-known members of this family are E-selectin and P-selectin, which both primarily appear on vascular endothelial cells. Of particular interest to this study is the evidence that selectins can aid metastasis by binding onto hyperglycosylated circulating cancer cells, thereby promoting adhesion to the vascular endothelium, which eventually leads to tumor formation at secondary sites [39]. If LPAT agents can be used to coat circulating tumor cells and prevent their selectin-based adhesion, they would consequently be able to suppress the onset and progression of metastatic tumors (Fig. 4A).

As a first step for the cell-based assays, investigations looked at the effects of LPAT on the adhesive properties of the CRC cell lines. As a preliminary test, adhesion assays were performed using plates precoated with extracellular matrix proteins (sourced from FBS). From these results (Fig. 4B and C), several trends could be identified. With sialic acid-targeting **1** and **2**, differences in activities could be directly correlated with the relative expression of α2,3- and α2,6-linked sialic acids among the tested cell lines. For example, α2,6-linked sialic acid-targeting **2** was shown to be much more effective against cell lines that produce higher levels of α2,6-linked sialic acid. Against HCT-116, SW620, and LoVo, treatment with **2** could reduce adhesion by 34%, 52%, and 58%, respectively, which are lower values than the ones obtained using **1**. Conversely, the α2,3-linked sialic acid-targeting **1** was more effective against cell lines that produce higher levels of α2,3-linked sialic acid. Against DLD-1, only treatment by **1** reduced adhesion at a significant level (42%). When looking at the adhesion obtained using fucose-targeting **3**, the capacity to impair cell adhesion correlated well with fucose expression levels. Against high-fucose-expressing cell lines, treatment with **3** was observed to reduce adhesion by roughly 34% (LoVo), 60% (HCT-116), and 71% (SW620). It should be noted that against a normal colon cell line (NCM-460), treatment with LPAT **1** to **3** was never observed to elicit any antiadhesive properties. Furthermore, the lectin-deficient LPAT **4** was never capable of impairing cancer cell adhesion (Fig. 4D), thereby confirming glycan binding to be a major factor in driving the antiadhesive effects caused by LPAT.

By summarizing the acquired adhesion assay results with the glycan/MMP9 profiles of each tested cell line (Fig. S4), a major factor in determining LPAT efficacy can be linked to the expression of the targeted glycans. For example, the 2,3-sialic acid-targeting LPAT **1** was found to be the most effective against DLD-1, which is the cell line that produces the most 2,3-sialic acid in this study. Likewise, the 2,6-sialic acid-targeting LPAT **2** impaired the adhesion of LoVo and SW620 the most, which are the top 2 cell lines with the highest levels of 2,6-sialic acid. Finally, the fucose-targeting LPAT **3** was observed to be the most effective against HCT-116 and SW620, which are the cell lines with the highest detectable levels of fucose in this study. Considering these preliminary experiments and all other factors (metastasis maps, Dukes cancer profiling, glycan/MMP9 profiling experiments, and correlation coefficients between cell targetability and LPAT activity), HCT-116 was chosen as the model cell line going forward as it represents the best in vitro model to gauge the efficacy of LPAT against colorectal metastasis in subsequent experiments. In addition, literature evidence also shows that HCT-116 can be highly metastatic in mouse models [40].

To next gauge the ability of LPAT agents at preventing cancer cell binding to selectins, cell adhesion assays were carried out using plates precoated with recombinant human P-selectin (Fig. 4E) and E-selectin (Fig. 4F). In general, all tested LPAT agents **1** to **3** exhibited significant inhibitory activity on the adhesive properties of HCT-116. However, the reduction of cell adhesion was the most pronounced with **3**-treated cells on E-selectin-coated plates, which experienced nearly complete suppression. In terms of the controls, uncleavable **1n** to **3n** variants and the nontargeting **4** were all observed to have negligible impact on impairing cell adhesion to P/E-selectin plates. Moving forward, the ability of **3** to prevent E-selectin-based cellular interactions was an important observation as literature studies have long recognized that highly metastatic colonic carcinoma cells bind E-selectin-expressing endothelial cells with much higher affinities [41]. Thus, the rationale of using multivalent LPAT complexes to block crucial E-selectin-based adhesion events in the metastatic process presents itself as a viable approach.

**Fig. 5.**
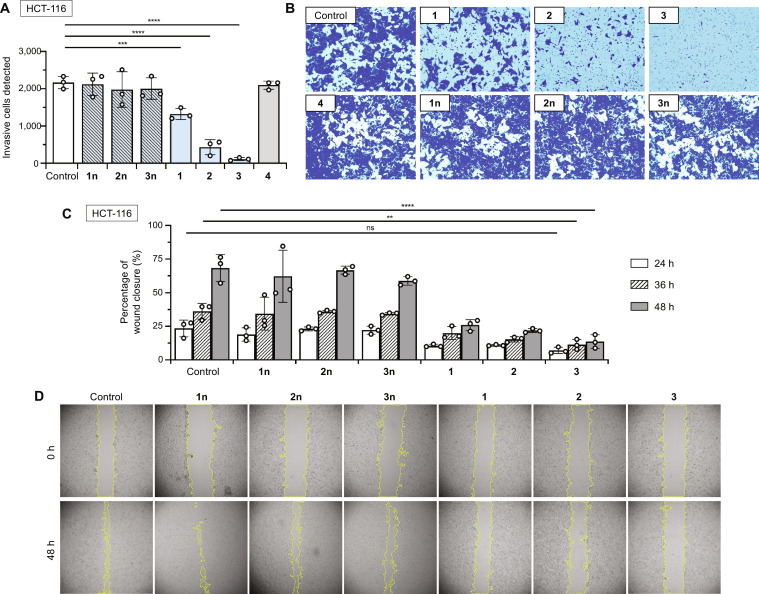
Anti-invasive and antimigratory properties of lectin-directed protein aggregation therapy (LPAT) agents 1 to 3. (A and B) Invasion assays were carried out through treatment with LPAT agents 1 to 3 and their related controls (10 μM) for 24 h on HCT-116 cells. After treatment, cells were seeded onto Matrigel-coated transwell chambers to investigate their invasiveness, which can be quantified by the number of invading cells (stained by crystal violet) found on images (at 10× magnification) of the lower insert membranes. (C and D) Wound healing assays were done by treatment with LPAT agents 1 to 3 and their related controls (10 μM) for various time points on HCT-116 cells. The degree of cell migration can be determined by measuring the wound gap area found on images (at 5× magnification) taken over various time points. All numerical data are presented as the mean ± standard error of the mean (SEM) of 3 replicates. Statistical analysis was performed using a one-way analysis of variance (ANOVA) with Tukey’s multiple comparisons test. *P < 0.03; **P < 0.002; ***P < 0.0002; ****P < 0.0001; ns, not significant.

In the next part, investigations were made into the ability of LPAT to affect the invasiveness of CRC cells. Conducted using standard cell invasion assay protocols, LPAT agents **1** to **3** were incubated with HCT-116 cells and then observed for any inhibitory effects on cell invasion (Fig. 5A and B and Fig. S5). Under the control conditions, the highly invasive HCT-116 cell line was found to easily migrate to the lower membrane of Matrigel-coated transwells (average cell count of ~2,170 in obtained images). However, once exposed to treatment conditions, detected counts of invasive HCT-116 cells dropped by 39% with **1**, 80% with **2**, and 95% with **3**, thereby proving effective inhibition of cell invasion. When testing the controls, the uncleavable **1n** to **3n** variants and the nontargeting **4** were all found to have negligible impact on impairing cell invasion.

Investigations were then made into the ability of LPAT to affect the migration of CRC cells. Following standard cell migration assay procedures, LPAT agents **1** to **3** were incubated with HCT-116 cells under varying conditions to judge the inhibitory effects on a cell’s capacity to close a wound gap (Fig. 5C and D and Fig. S6). Based on the control data, 68% of the wound could be closed following a recovery time of 48 h. However, LPAT treatment at the same time point was observed to significantly suppress wound recovery, as only 21%, 30%, and 14% of the wound could be closed following treatment with **1**, **2**, and **3**, respectively. As a control, the uncleavable **1n** to **3n** variants and the nontargeting **4** were all tested and found to have no significant impact on preventing wound closure. As a further test to validate the mechanism of LPAT, cell migration assays were also conducted on HT-29 cells, which are considered in this study to have lower expression levels of fucose and MMP9 (Figs. S7 and S8). From these data, no significant changes in wound healing could be observed following LPAT **1** to **3** treatment. Thus, it can be strongly suggested that the antimigratory activities of LPAT are largely confined to cell lines that fit a specific glycan/MMP9 expression profile. Collectively from these cell-based assays, it can be clearly observed that LPAT agents, particularly the fucose-targeting **3**, possessed strong antiadhesive, anti-invasive, and antimigration activity against HCT-116 cells.

**Fig. 6.**
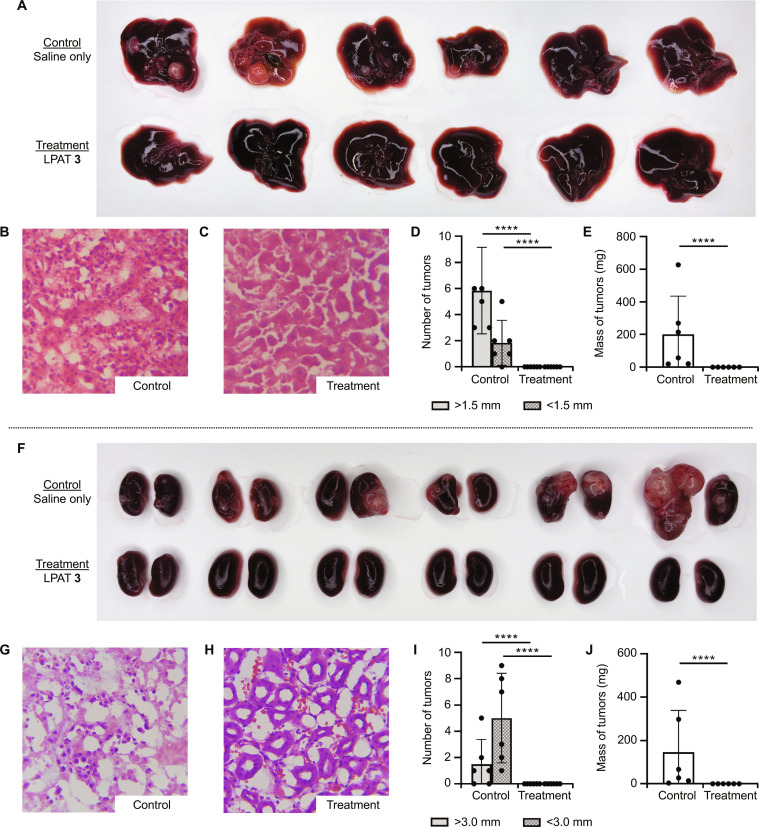
Exploring the anti-metastatic properties of lectin-directed protein aggregation therapy (LPAT) agent 3 in mice (via an experimental metastasis model). To induce metastatic tumors, HCT-116 cells were injected into mice through the tail vein. Two groups were then created; the treatment group (*n* = 6) received a follow-up injection of LPAT 3 at a dosage of 12 mg/kg, while the control group (*n* = 6) instead received a shot of saline. Following 8 weeks, all mice were euthanized and autopsied for tumor burden. For both the control and treatment groups, images of the extracted (A) liver and (F) kidney tissues are shown. Histological analysis of the control group confirms the development of tumors in the (B) liver and (G) kidney. Histological analysis of the treatment group confirms healthy (C) liver and (H) kidney tissues. The total number of individual tumors (per mouse) originating from (D) liver and (I) kidney tissues were counted, measured, and grouped based on size. The collective mass of tumors (per mouse) originating from (E) liver and (J) kidney tissues was weighed and recorded. Statistical analysis was performed using an unpaired *t* test. All numerical data are presented as mean ± standard error of the mean (SEM). *P < 0.03; **P < 0.002; ***P < 0.0002; ****P < 0.0001; ns, not significant.

To evaluate the therapeutic potential of LPAT against metastatic CRC, studies were next carried out using an experimental metastasis mouse model (Fig. 6). To establish this model, HCT-116 cells can be directly introduced into the circulatory system of NSG mice via intravenous tail vein injections. After a roughly 8-week period, these models were shown to consistently develop liver and kidney tumors, thereby mimicking the metastatic process. To gauge the bioactivity of LPAT, mice injected with HCT-116 cells were quickly arranged into control and treatment groups, followed by a second injection round. The mice in the control group received a saline solution, while the mice in the treatment group received a dosage of 12 mg/kg of LPAT **3**. After 8 weeks, all mice were sacrificed and then autopsied for liver and kidney tumors.

Figure 6A shows the images of extracted liver samples from both control and treatment groups. By observation, it is clear to see that the control mice experienced significantly a higher tumor burden than the treatment group, which can be corroborated by histology (Fig. S9A). Tissue slices from prominent liver tumors in the control group showed clear signs of cancer pathology, such as multinucleated hepatocytes (Fig. 6B). On the other hand, liver slices obtained from the treatment group exhibited a healthy hepatocyte structure since these cells were shown to be arranged into cords separated by vascular sinusoids (Fig. 6C). To better quantify tumor burden, tumor nodules were individually extracted and counted (Fig. 6D and Fig. S10A). Within the control group, both large (>1.5 mm) and small (<1.5) tumors were counted in significant excess compared to those in the treatment group. These extracted tumors were also collectively weighed, once again highlighting the higher tumor burden in the control group (Fig. 6E).

Similarly, Fig. 6F shows the images of extracted kidney samples from both control and treatment groups. Again, histological analysis confirmed that control mice consistently experienced a higher tumor burden compared to the treatment group (Fig. S9B). For example, tissue slices from kidney tumors in the control group showed irregular cell shapes with more transparent cytoplasms. In addition, cells appeared to be multinucleated, with most of the nuclei adopting irregular shape patterns (Fig. 6G). However, kidney slices from the treatment group instead showed the usual simple cuboidal epithelium structures commonly found in kidney tubules (Fig. 6H). In a similar effort to quantify tumor burden, tumor nodules were again extracted and counted (Fig. 6I and Fig. S10B). In this case, the number of small (<3.0 mm) tumors exceeded the number of larger (>3.0 mm) tumors. Yet, the number of total tumors found in the kidneys of the control group were still counted in significant excess compared to those in the treatment group. The collective weight of the extracted kidney tumors was again much higher in the control group (Fig. 6J).

Overall, the animal studies were able to validate that targeting hyperfucosylation by LPAT **3** could be a viable strategy for preventing the metastatic spread of CRCs. When LPAT treatment was applied, mice were consistently found to have the onset and growth of liver and kidney tumors significantly suppressed compared to those in controls.

## Conclusion

CRC is widely regarded as one of the most commonly diagnosed cancers worldwide, with some metrics anticipating rates of 3.2 million new cases and 1.6 million deaths by 2040 [42]. A key reason for the lethality of CRC is its high rate of metastasis, which sees about 20% of patients already carrying metastases at initial times of CRC diagnosis. Considering this, it would be highly beneficial to develop drugs that can target and suppress the metastatic tumor spread of CRC, effectively functioning as a preventative therapy.

Recently, our group has developed a therapy based on the concept of cancer-activated lectin multivalency [25], which relies on tumor-associated proteases to elicit the formation of protein complexes that bind strongly to metastatic cancer cells through lectin multivalency. In the context of metastatic CRC, our study found that targeting hyperfucosylation was the most effective. These results largely corroborate with literature studies that have shown the metastatic potential of CRC to be linked with the up-regulation of several FUTs, such as FUT1 [43], FUT2 [44], FUT3 [45], FUT5 [46], FUT6 [46], FUT7 [47], and FUT9 [48]. Furthermore, highly metastatic colonic carcinoma cells are also known to express more (fucosylated) sialyl Lewis X structures than their lower metastatic counterparts [49].

**Fig. 7.**
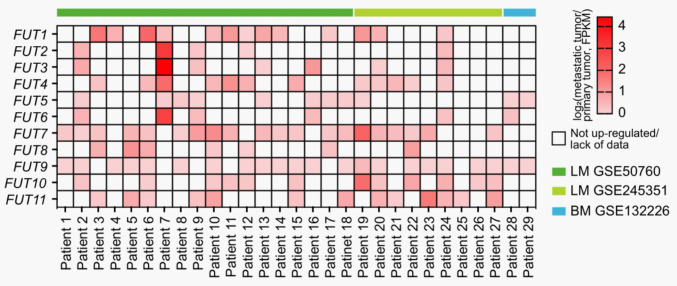
Analysis of fucosyltransferase gene expression in paired (primary tumor and metastases) clinical isolates obtained from 29 different patients. The heatmap was generated to highlight the general up-regulation of fucosyltransferase 1 (FUT1) to fucosyltransferase 11 (FUT11) genes in liver or brain metastatic tumors compared to their primary colorectal tumor counterparts. Red represents gene up-regulation, while white represents genes that are not up-regulated or where data are unavailable.

To further strengthen the argument that hyperfucosylation plays an important role in the metastatic process of CRCs, comparative gene expression profiling was conducted on RNA sequencing data obtained from clinical metastatic colorectal tumor samples. When looking at paired (primary tumor and metastases) clinical isolates obtained from 29 different patients, a general increase in FUT activity can be observed (Fig. 7). For example, 13 of the patients saw increased expression in >5 of their FUT genes, while 15 patients experienced increases between 2 and 4 of their FUT genes. While there appears to be no FUT isoform that is absolutely up-regulated, FUT7 (20 out of 29 cases) and FUT9 (22 out of 29 cases) were found to be consistently up-regulated in most of the patients. This analyzed connection between increased fucosylation to the metastatic process can also be supported by a previous study, which directly examined 50 clinical samples from CRC patients [50]. Considering these results, there is a good possibility that LPAT could be effective against clinical metastasis.

Overall, this work succeeded in adapting LPAT toward metastatic CRCs. Through screening different lectins that bind to different forms of hypersialylation and hyperfucosylation, it was determined that LPAT was most effective when targeting hyperfucosylated/MMP9-overexpressing CRC cells. For example, treatment of the HCT-116 cell line exhibited significant reductions in cellular adhesion, invasion, and migration. These results are highly encouraging for future drug development as human-derived HCT-116 cells have been shown repeatedly throughout the literature to be highly metastatic in mouse models [40]. While it is anticipated that this work can possibly form the foundation of a metastatic CRC prevention therapy, it should be acknowledged that several key tests need to be addressed in future preclinical experiments. For example, there is currently no data to directly prove the specific binding of the activated LPAT complexes onto metastatic cancer cell surfaces. To address this issue, LPAT agents will need to be modified with a small molecule fluorophore on either the aggregating unit or targeting lectin in a site-selective manner. Testing then needs to confirm that LPAT’s functional capabilities (i.e., self-assembly or glycan binding) are not impaired before further experimentation. Another important preclinical task will be to conduct rigorous screening of the off-targeting effects of the LPAT system against other cell lines of normally fucosylated tissues (i.e., intestinal stem cells and blood group O erythrocytes). These examples, along with a host of other experiments, will need to be completed to ensure that LPAT exhibits potent anti-metastatic activity while possessing a good overall safety profile.

## Data Availability

All data supporting the findings of this study are included in the published article and the Supplementary Materials. Data can also be requested from the corresponding authors upon reasonable request.
