# Supplementary material for: Lectin-Directed Protein Aggregation Therapy toward Hyperfucosylated and Hypersialylated Metastatic Colorectal Cancers
Source: Biomater Res. 2026 Jul 17;30:0394. doi: 10.34133/bmr.0394 (PMC13376382; doi:10.34133/bmr.0394)
Supplement: Supplementary 1 — Figs. S1 to S10 [file bmr.0394.f1.pdf]

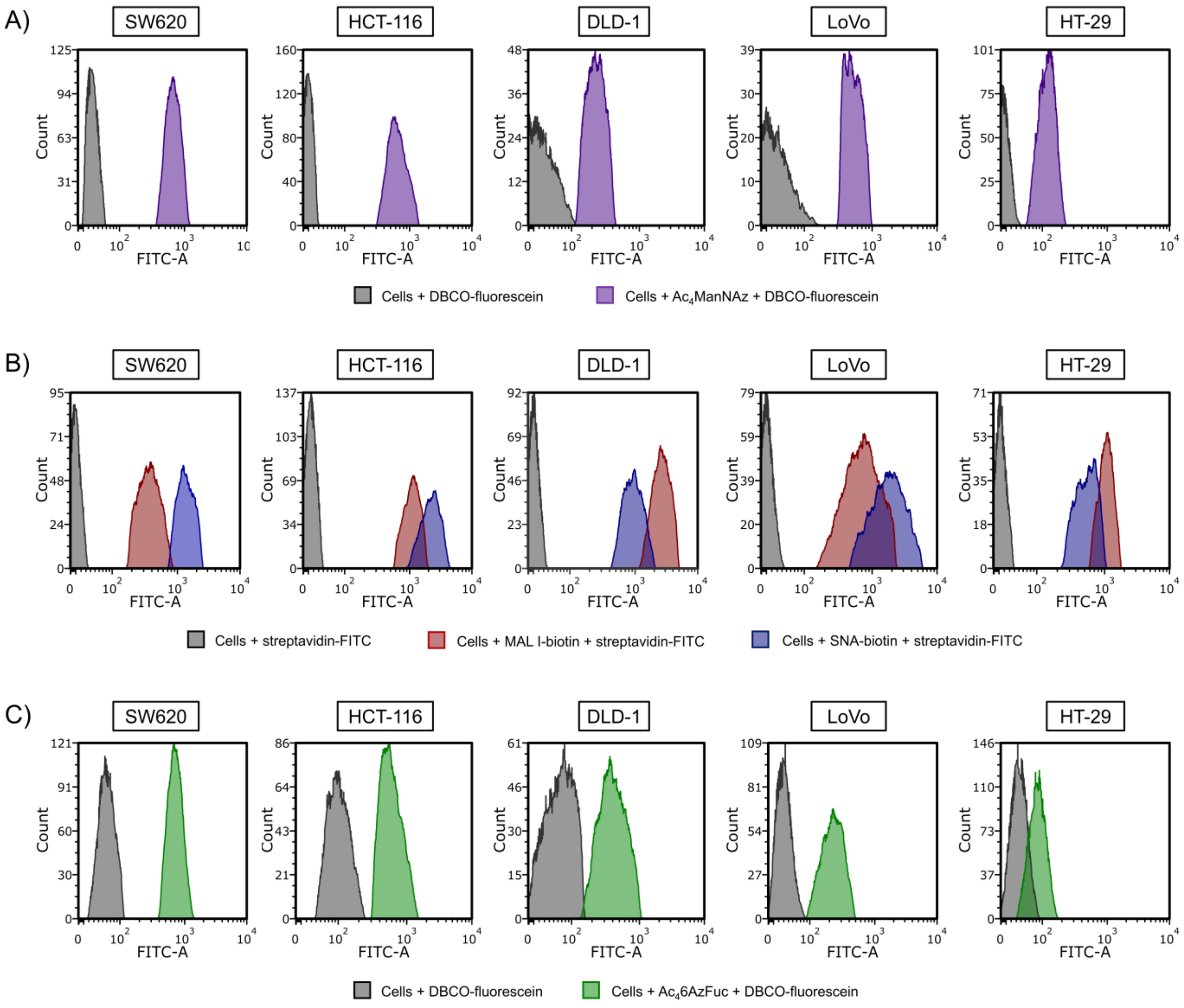

D)

	Total sialic acid levels (metabolic labelling)		α2,3- and α2,6- sialic acid levels (lectin labelling)			Total fucose levels (metabolic labelling)	
**Cell line**	**Cells + Ac$_4$ManNAz + DBCO-FITC**	**Cells + DBCO-FITC**	**Cells + MAL I-biotin + SAV-FITC**	**Cells + SNA-biotin + SAV-FITC**	**Cells + SAV-FITC**	**Cells + Ac$_4$6AzFuc + DBCO-FITC**	**Cells + DBCO-FITC**
SW620	509.03<br>606.04<br>703.05	15.26<br>16.35<br>20.71	369.82<br>314.88<br>329.64	971.70<br>786.38<br>1083.22	5.74<br>3.28<br>4.92	772.20<br>678.48<br>572.88	59.84<br>55.44<br>52.80
HCT-116	457.56<br>528.90<br>687.98	5.74<br>5.74<br>7.38	788.02<br>974.98<br>1195.56	1560.46<br>1781.86<br>2288.62	11.48<br>11.48<br>12.30	692.52<br>611.61<br>777.78	77.43<br>85.26<br>102.23
DLD-1	309.72<br>231.42<br>170.52	8.70<br>3.48<br>3.48	1946.19<br>2513.43<br>2972.79	450.66<br>515.91<br>580.72	6.96<br>6.96<br>7.83	408.03<br>289.71<br>212.28	102.66<br>73.95<br>52.20
LoVo	762.99<br>548.97<br>382.36	12.18<br>7.83<br>2.61	429.78<br>637.28<br>855.65	982.23<br>1470.74<br>1779.15	6.09<br>7.83<br>13.05	349.30<br>284.49<br>278.40	53.07<br>30.45<br>11.31
HT-29	144.42<br>100.92<br>80.91	2.61<br>3.48<br>0.87	1098.81<br>1014.42<br>862.17	580.72<br>764.73<br>748.2	11.31<br>9.57<br>8.70	100.05<br>78.30<br>64.38	40.02<br>30.45<br>25.23

Figure S1. Flow cytometry histograms that were obtained to determine A) total levels of cell surface exposed sialic acid, B) relative expression levels of α2,3- and α2,6-linked sialic acid, and C) total levels of cell surface exposed fucose. D) Summary of the replicate data obtained from FACS analysis for the observed mean fluorescence intensities of the cell populations under study.

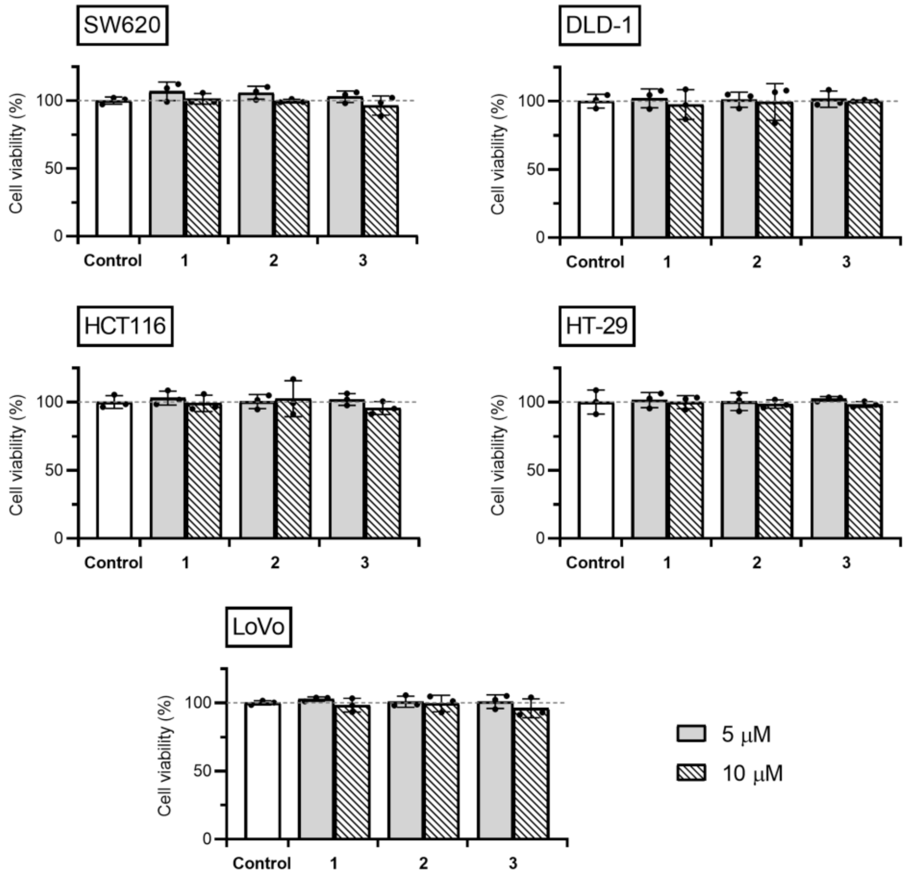

Figure S2. Short-term (1-day) cell viability tests to evaluate the cytotoxicity of LPAT agents **1**-**3** against the colorectal cancer cell lines under study (SW620, DLD-1, HCT116, HT-29, LoVo). All numerical data is presented as a mean ± s.e.m. of three replicates. Statistical analysis was performed using a one-way ANOVA with Tukey's multiple comparisons test. *P<0.03, **P<0.002, ***P<0.0002, ****P<0.0001, ns = not significant.

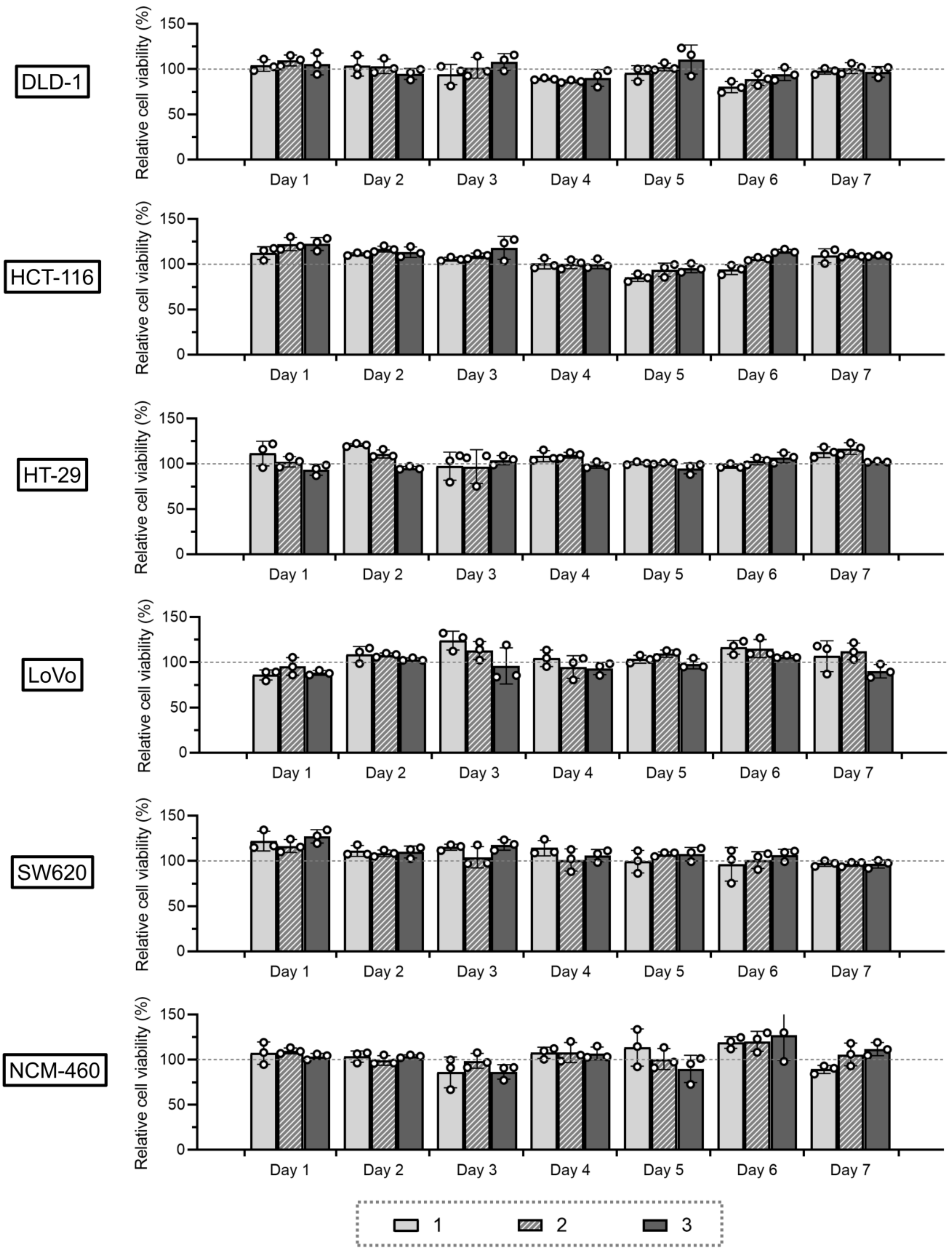

Figure S3. Long-term (7-day) cell viability tests to evaluate the cytotoxicity of LPAT agents **1**-**3** (10 μM) against the cell lines under study (SW620, DLD-1, HCT116, HT-29, LoVo, NCM-460). All numerical data is presented as a mean ± s.e.m. of three replicates. Statistical analysis was performed using a one-way ANOVA with Tukey's multiple comparisons test. *P<0.03, **P<0.002, ***P<0.0002, ****P<0.0001, ns = not significant.

A)

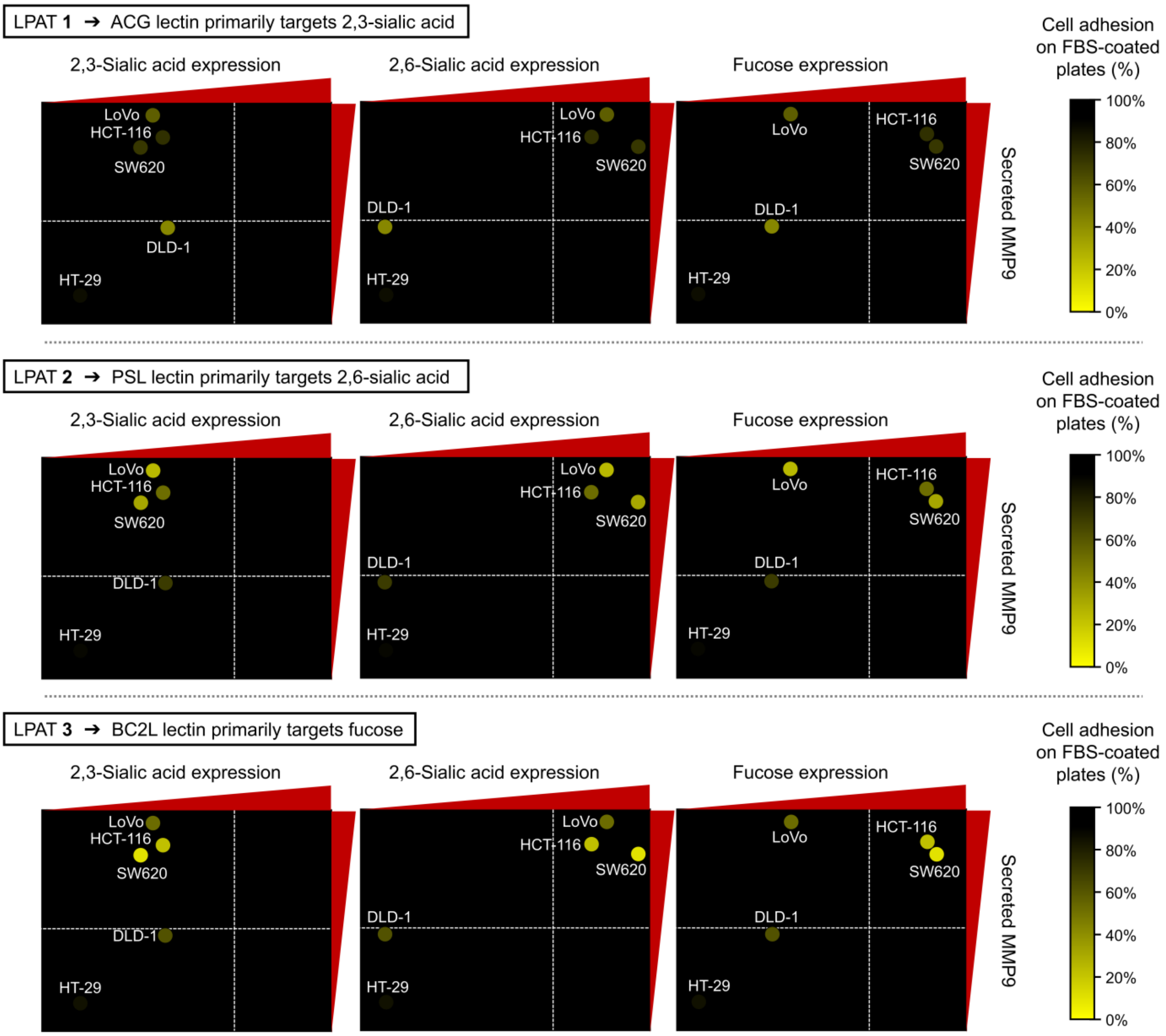

B)

Cell line	Targetability score[(a)]	Percentage of inhibition by LPAT **1**	Correlation coefficient
LoVo	0.901	29.3	
HCT-116	0.891	14.9	
SW620	0.790	17.0	0.5047
DLD-1	0.699	42.3	
HT-29	0.216	3.9	

[(a)] based on MMP9/2,3-sialylation profile

Cell line	Targetability score[(b)]	Percentage of inhibition by LPAT **2**	Correlation coefficient
LoVo	0.895	57.8	
HCT-116	0.820	34.0	
SW620	0.889	52.1	0.9437
DLD-1	0.262	18.5	
HT-29	0.109	2.0	

[(b)] based on MMP9/2,6-sialylation profile

Cell line	Targetability score[(c)]	Percentage of inhibition by LPAT **3**	Correlation coefficient
LoVo	0.663	33.6	
HCT-116	0.850	60.1	
SW620	0.848	70.9	0.9497
DLD-1	0.380	25.6	
HT-29	0.096	5.1	

[(c)] based on MMP9/fucosylation profile

Figure S4. A) Collection of charts to map the effectiveness (anti-adhesive capabilites) of either LPAT **1**, **2**, or **3** with the glycan/MMP9 profiles of the varying colorectal cancer cell lines under study. Yellow spots are used to visualize maximal cell adhesion impairment, while black spots are used to visualize minimal effects to cell adhesion. Chart backgrounds are also colored black to better highlight affected cell lines. For the charts of each LPAT agent, the horizontal axes represent the levels of detected sialic acid (either 2,3- or 2,6-linked) or fucose expresion for each cell line. The vertical axes represent the leves of secreted MMP9 for each cell line. B) Correlation coefficients were calculated to determine the relationship between cell lines with good targetability (high MMP9 and glycosylation levels) and their susceptibility to the anti-adhesive activities of related LPAT agents.

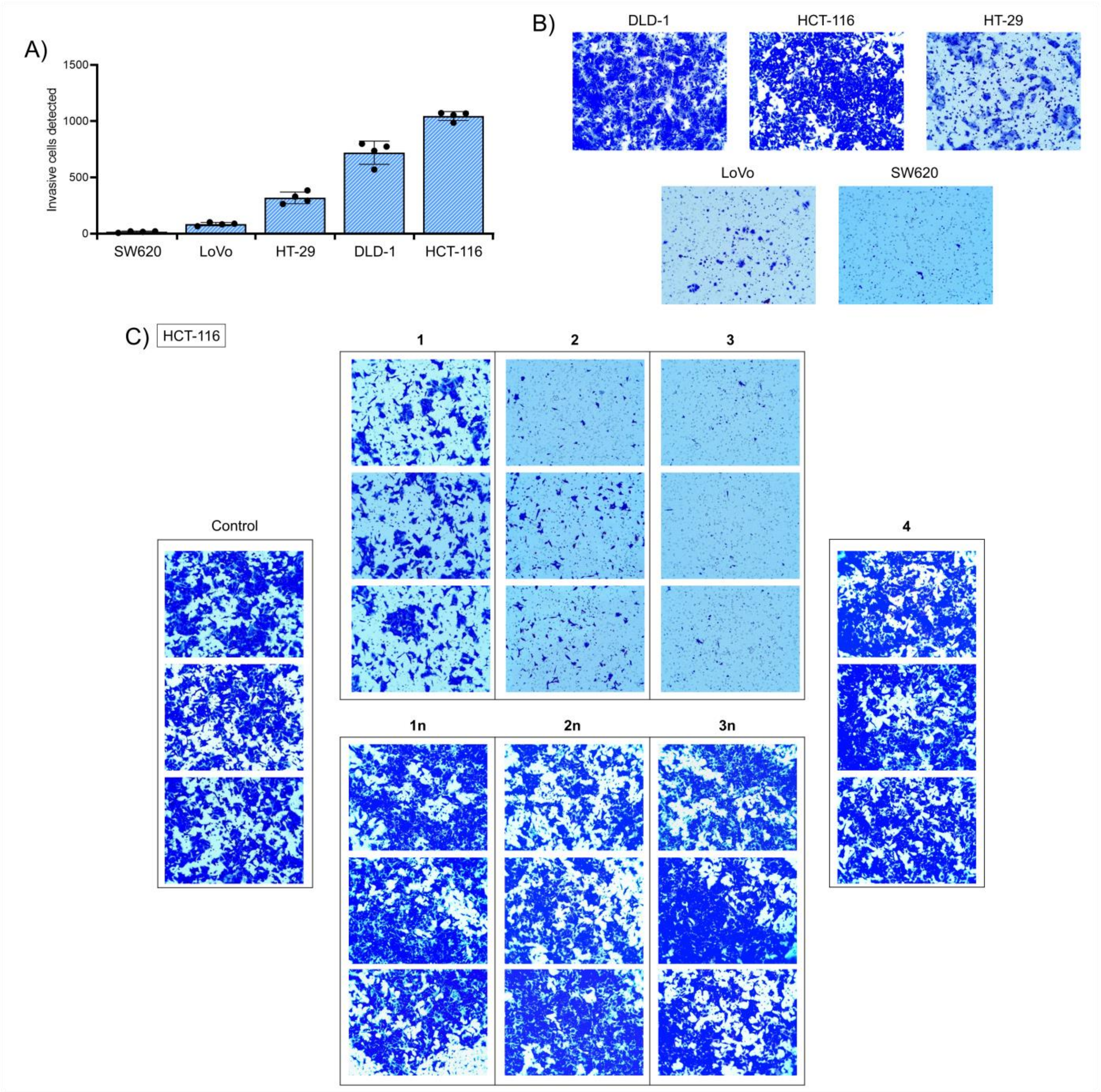

Figure S5. Detailed data for the cell invasion assay. A-B) As a preliminary test, the comparative invasiveness of the colorectal cancer cell line panel was explored. To do this, each cell line was seeded at about $1{\times}10^5$ cells onto matrigel-coated transwell chambers, followed by incubation for 24 hr at 37°C. Invading cells on the lower insert membranes were then stained (with crystal violet), imaged using a microscope at 10× magnification, and quantified. C) Invasion assays were carried out on HCT-116 cells by first treating cells to the various LPAT agents (10 μM) for 24 hours. Afterwards, cells were seeded onto matrigel-coated transwell chambers, followed by incubation for 24 hr at 37°C. Invading cells on the lower insert membranes were then stained (with crystal violet), imaged using a microscope at 10× magnification, and quantified. Shown in this figure are the images obtained for analysis. All numerical data is presented as a mean ± s.e.m. of three replicates.

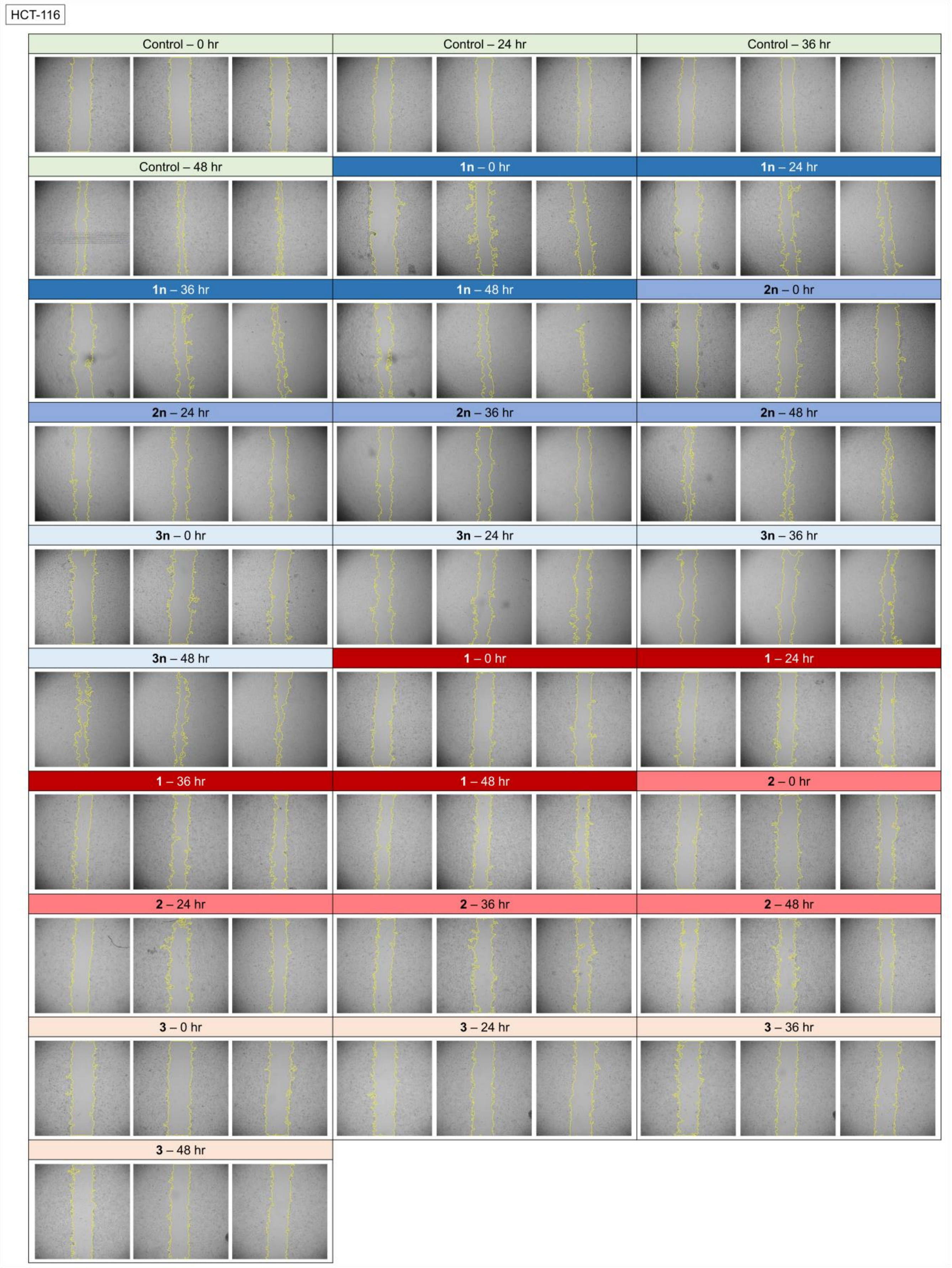

Figure S6. Images obtained from the cell migration assays involving the treatment of varying LPAT agents with HCT-116 cells. After wound initation and treatment, cell images were obtained at specific time intervals (0, 24, 36, and 48 hr) using a microscope at 5× magnification.

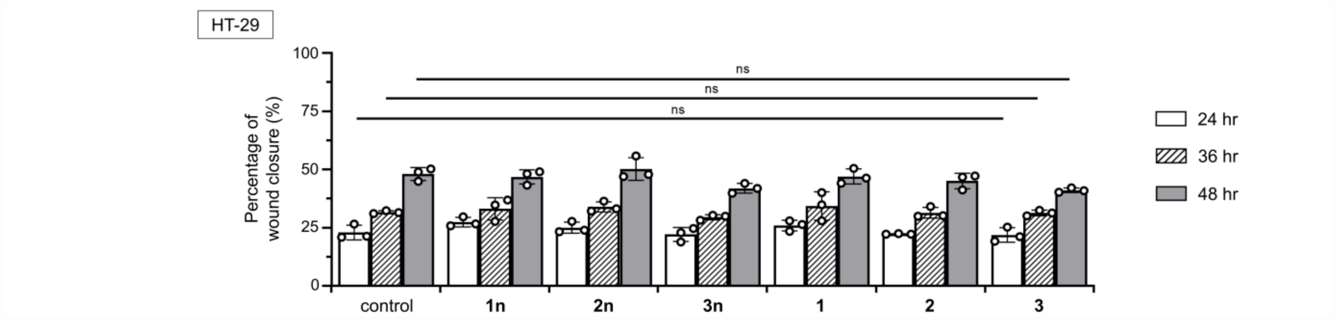

Figure S7. Summary of cell migration assay data for the incubation of varying LPAT agents (10 μM) with HT-29 cells. At specific time intervals in this experiment, cell images were obtained using a microscope to quantify the percentage of wound closure. This calculation is based on the measurment of the wound area at various time points, followed by its comparison to the wound area of the control group (no treatment). All numerical data is presented as a mean ± s.e.m. of three replicates. Statistical analysis was performed using a one-way ANOVA with Tukey's multiple comparisons test. *P<0.03, **P<0.002, ***P<0.0002, ****P<0.0001, ns = not significant.

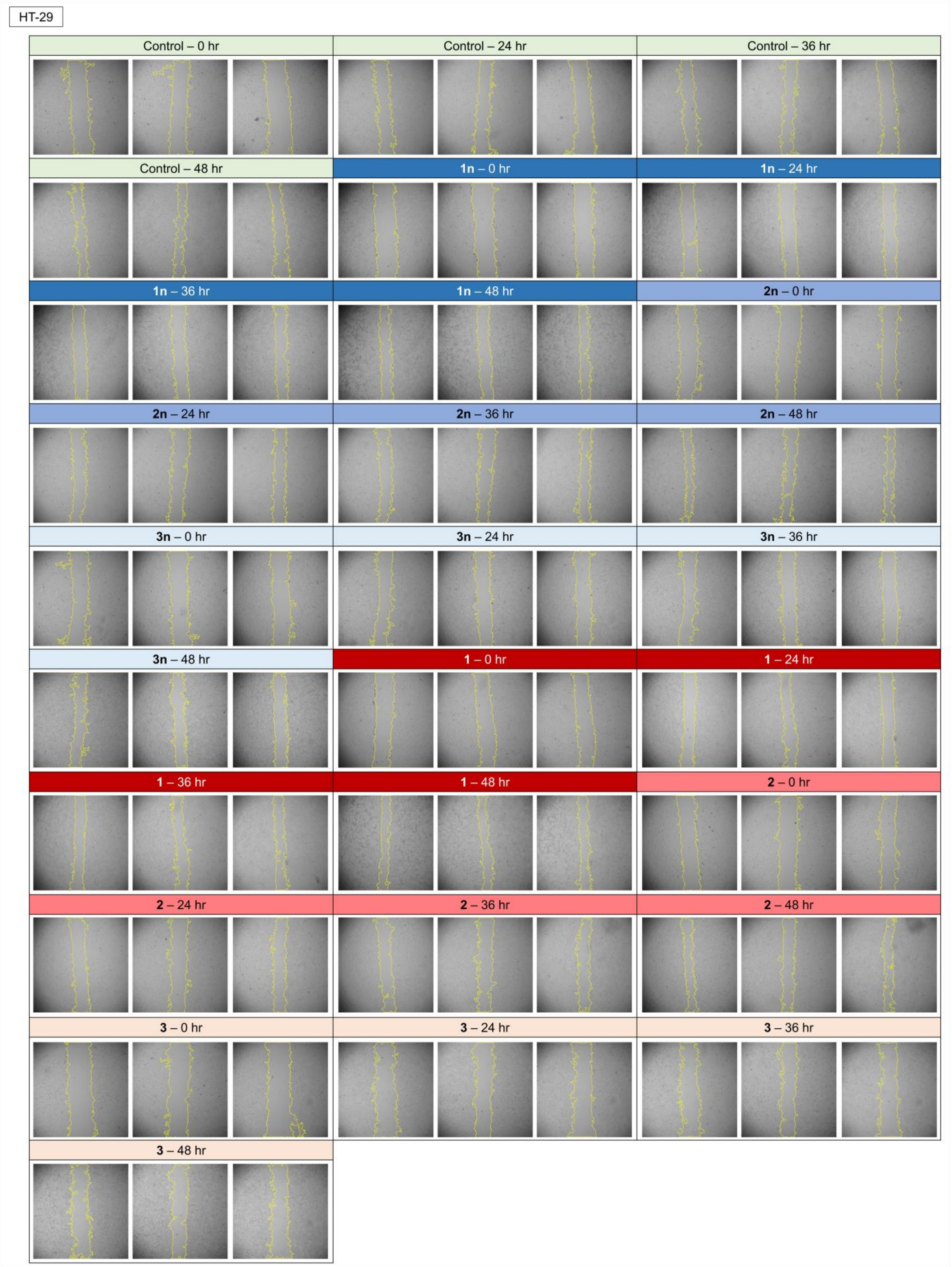

Figure S8. Images obtained from the cell migration assays involving the treatment of varying LPAT agents with HT-29 cells. After wound initation and treatment, cell images were obtained at specific time intervals (0, 24, 36, and 48 hr) using a microscope at 5× magnification.

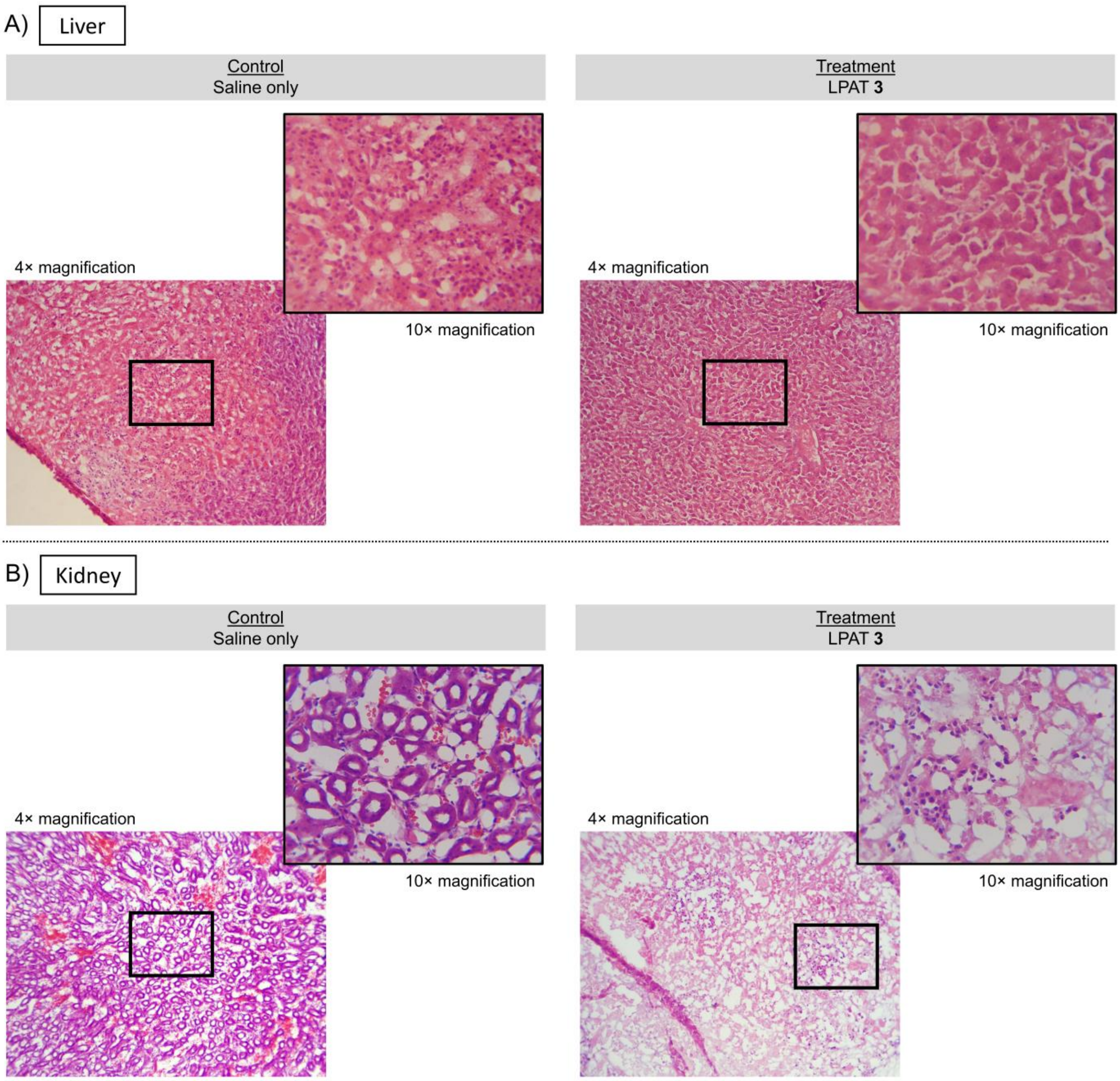

Figure S9. Representative images of H&E-stained samples (4× and 10× magnification) showing the histological features of tissues extracted from mice of either the control or treatment groups. With a focus on metastatic tumors developed by HCT-116 cells injected into mice, histological analysis focused on A) liver, and B) kidney tissues. Sampling was done on organs extracted from the six mice of each group.

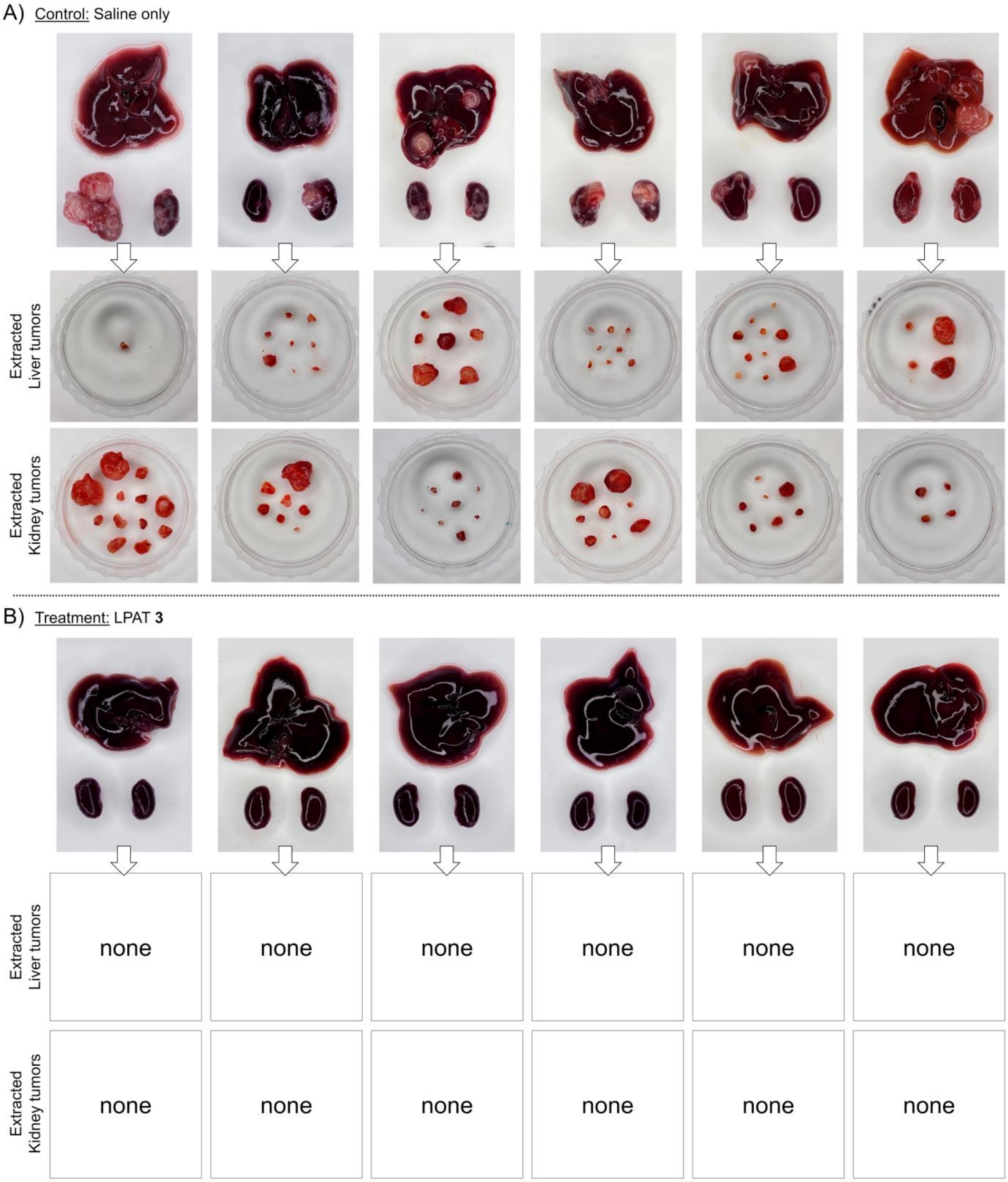

Figure S10. Detailed images obtained from the LPAT treatment experiments using the spontaneous metastasis mouse model. A) Images of the whole liver and kidneys extracted from individual mice (n = 6) of the control group. B) Images of the whole liver and kidneys extracted from individual mice (n = 6) of the treatment group. For both the control and treatment groups, organs were lightly cut to excise any (large or small) tumors growing on the outside or within the organs. These tumors were then collected, counted, weighed. Accompanying images show the collected liver and kidney tumors extracted from each individual mouse.
